# Case Report: A large granular cell tumor of the cervical esophagus with single cell RNA sequencing analysis

**DOI:** 10.3389/fonc.2025.1580121

**Published:** 2025-09-02

**Authors:** Chengying Shao, Keyu Chen, Yanting Duan, Jiajie Xu

**Affiliations:** ^1^ Second Clinical Medical College, Zhejiang Chinese Medical University, Hangzhou, China; ^2^ Otolaryngology & Head and Neck Center, Cancer Center, Department of Head and Neck Surgery, Zhejiang Provincial People’s Hospital (Affiliated People’s Hospital), Hangzhou Medical College, Hangzhou, Zhejiang, China; ^3^ Zhejiang Key Laboratory of Precision Medicine Research on Head & Neck Cancer, Zhejiang Provincial People’s Hospital (Affiliated People’s Hospital), Hangzhou Medical College, Hangzhou, Zhejiang, China; ^4^ Zhejiang Provincial Clinical Research Center for Head & Neck Cancer, Zhejiang Provincial People’s Hospital (Affiliated People’s Hospital), Hangzhou Medical College, Hangzhou, Zhejiang, China

**Keywords:** granular cell tumor, single-cell RNA sequencing, myocutaneous free flap, esophageal tumor, esophageal reconstruction

## Abstract

Esophageal granular cell tumor (GCT) is a rare benign neurogenic tumor, however, malignant transformation has been reported. And there is no consensus on the choice of esophageal reconstruction in these patients. Therefore, the study of markers of malignant potential in GCT is of great importance in guiding the choice of clinical treatment. Case presentation: Herein, we report the case of a patient with a large cervical esophageal GCT in which a myocutaneous free flap was used to repair a large defect after the resection of a localized esophageal tumor, providing strong reliability in terms of coverage capacity, tissue resistance, and distance management from the recipient vessel. The patient’s recovery was satisfactory at 48 days postoperatively, without significant complications. To further understand the specific cellular status of esophageal granular cell tumors, we performed an in-depth analysis of the tumor and its paraneoplastic tissues in this patient using single-cell RNA sequencing (scRNA-seq), which showed that neural-like cell subpopulations were enriched in the tumor, and genes such as SOX10, S100B, NCAM1, SPP1, and STMN1 were significantly upregulated. A significant copy number variation increase was observed in the X chromosome region. Conclusions: To the best of our knowledge, the present study represents the first scRNA-seq analysis of GCT, providing valuable insights for future prediction of GCT malignancy. In addition, the present study successfully repaired a large cervical oesophageal defect using a skin flap, and these findings have great potential to guide the understanding and management of postoperative large defects in benign cervical esophageal masses, paving the way for further clinical surgical practice in this area.

## 1 Introduction

Granular cell tumors (GCT) are rare soft tissue tumors originating from Schwann cells and were first identified in the tongue by Abrikossoff in 1926 (1). GCT can occur in any part of the body, preferentially in the tongue, mammary glands, and proximal limbs, which are usually located in the skin, subcutaneous layer, or submucosal layer. GCT in the gastrointestinal tract are rare, accounting for only 8–10% of cases, with esophageal involvement observed in only 2% of cases, occurring primarily in the lower and middle esophagus (2–4), but only 20% and 15% of these are located in the middle and upper segments. Most GCTs are benign; however, tumors with atypical cytological features or malignant transformations have been reported (5, 6). Here, we share this case to offer a reference for clinically treating similar cases and to inspire related basic medical research.

## 2 Case presentation

**Figure 1:**
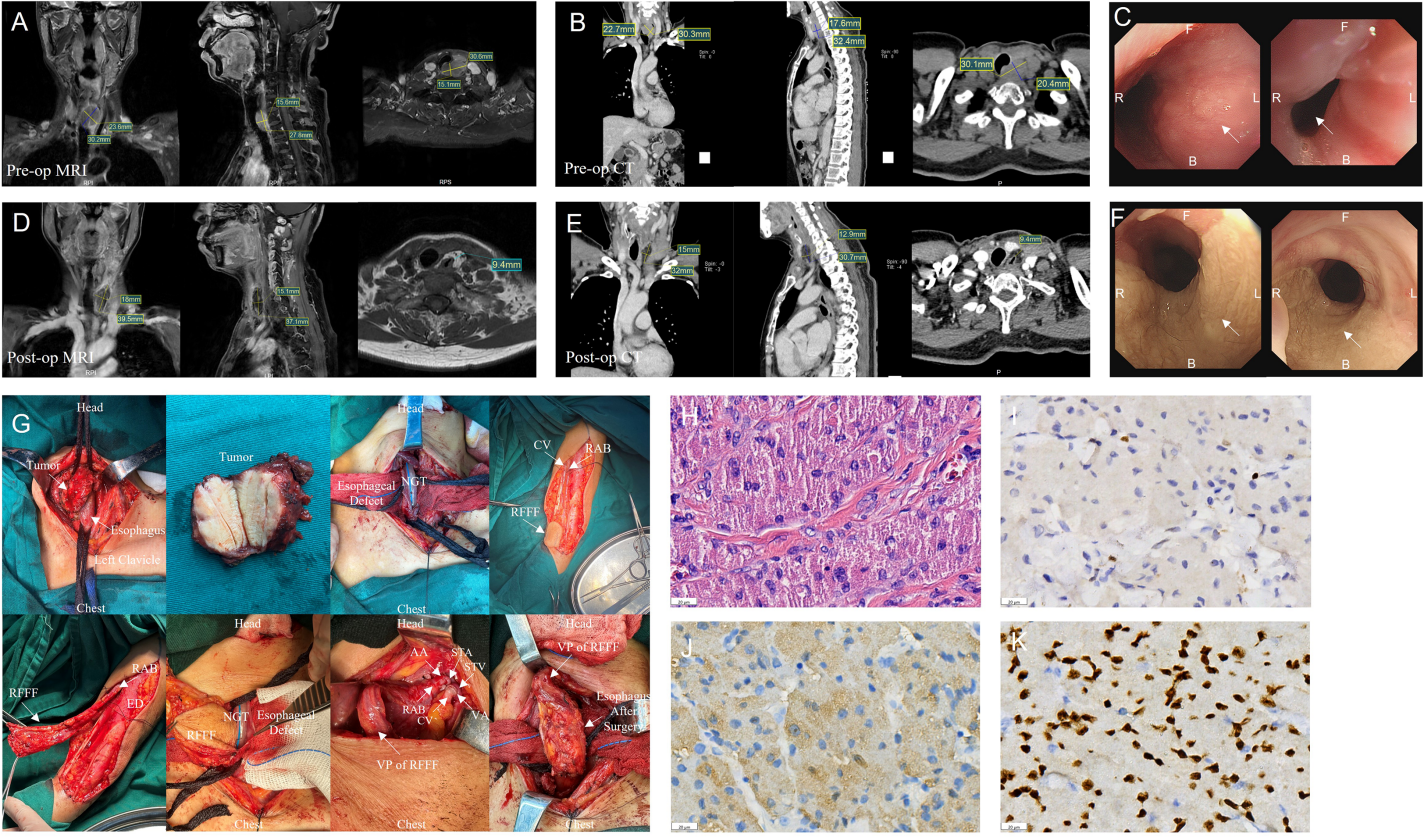
Information for patient. **(A-C)** Preoperative (pre-op) enhancement magnetic resonance imaging (MRI), enhanced computed tomography (CT) and Endoscopic examination results. **(D-F)** Postoperative (post-op) enhancement magnetic resonance imaging (MRI), enhanced computed tomography (CT) and Endoscopic examination results. **(G)** Surgery images. **(H)** Numerous eosinophilic granules visible within the cytoplasm under high magnification. Hematoxylin eosin staining; scale bar, 20 µm. **(I)** Tumor cells express Ki67 with about 3% in the hotspot. **(J)**. Positive expression of S100 in tumor cells. **(K)** Positive expression of SOX10 in tumor cells. (AA, Arterial anastomosis; B, back; CV, cephalic vein; ED, Extensor digitorum; F, Front; NGT, Nasogastric tube; RAB, radial arteriovenous bundle; RFFF, radial forearm free flap; STA, superior thyroid artery; STV, superior thyroid vein; VA, venous anastomosis; VP, Vascular pedicle).

A 56-year-old female with over-year swallowing difficulty was admitted. Her height, weight, and BMI were 155 cm, 52 kg, and 23.11 kg/m², respectively. Dysphagia, voice, and nutritional status were assessed using the Sydney Swallow Questionnaire (SSQ) (7), Dysphagia Outcome and Severity Scale (DOSS) (8), Voice Handicap Index (VHI) (9), Voice-Related Quality of Life (V-RQOL) (10) and Nutritional Risk Screening 2002 (NRS 2002), preoperatively and on postoperative day 50, respectively (**Table 1**). All laboratory test results were normal. Preoperative and postoperative day 50 Enhancement magnetic resonance imaging (MRI), Enhanced computed tomography (CT), and endoscopic examination are shown in **Figures 1A–F**. A preoperative needle biopsy indicated a GCT. Immunohistochemical showed that S-100 (+), SOX10 (+), P40 (–), P63 (-), CK (Pan) (-), Vimentin (+), Ki67 (+, 3%), PAS (+).

**Table 1:** Swallowing, voice, and nutritional assessment of the patient pre- and postoperatively.

Function	Scale	Abbreviation	Level/scores	
			Preoperative	Postoperative
Swallowing	Dysphagia Outcome and Severity	DOSS	Level 5	Level 7
	Sydney Swallow Questionnaire	SSQ	1095	90
Voice	Voice Handicap Index	VHI	15	3
	Voice-related Quality of Life	VRQOL	12	0
Nutrition	Nutritional Risk Screening 2002	NRS 2002	1	0

DOSS, Dysphagia Outcome and Severity Scale; NRS, Nutritional Risk Screening; SSQ, Sydney Swallow Questionnaire; VHI, Voice Handicap Index; VRQOL, Voice-Related Quality of Life.

After a multi-disciplinary discussion, a decision was made to perform partial cervical esophagectomy, radial forearm free flap (RFFF) grafting. The intraoperative observation is shown in **Figure 1G**. Routine histopathological examination of the esophageal tumor (**Figures 1H–K**) revealed a 3.2 × 3 × 1.3 cm GCT invading the esophageal subserosa without vascular or perineural invasion. Margins were negative, and no neoplasm invasion was observed. Immunohistochemistry results: S100(+), SOX10(+), P40(-), P63(-), CK(Pan) (-), Vimentin (+), Ki67(+), hot spot area about 3%, P53(+, varying intensity), PAS (+). The patient was discharged on day 20 after surgery with no complications like stenosis or fistula during follow-up.

## 3 Analytical results of single-cell RNA-seq

**Figure 2:**
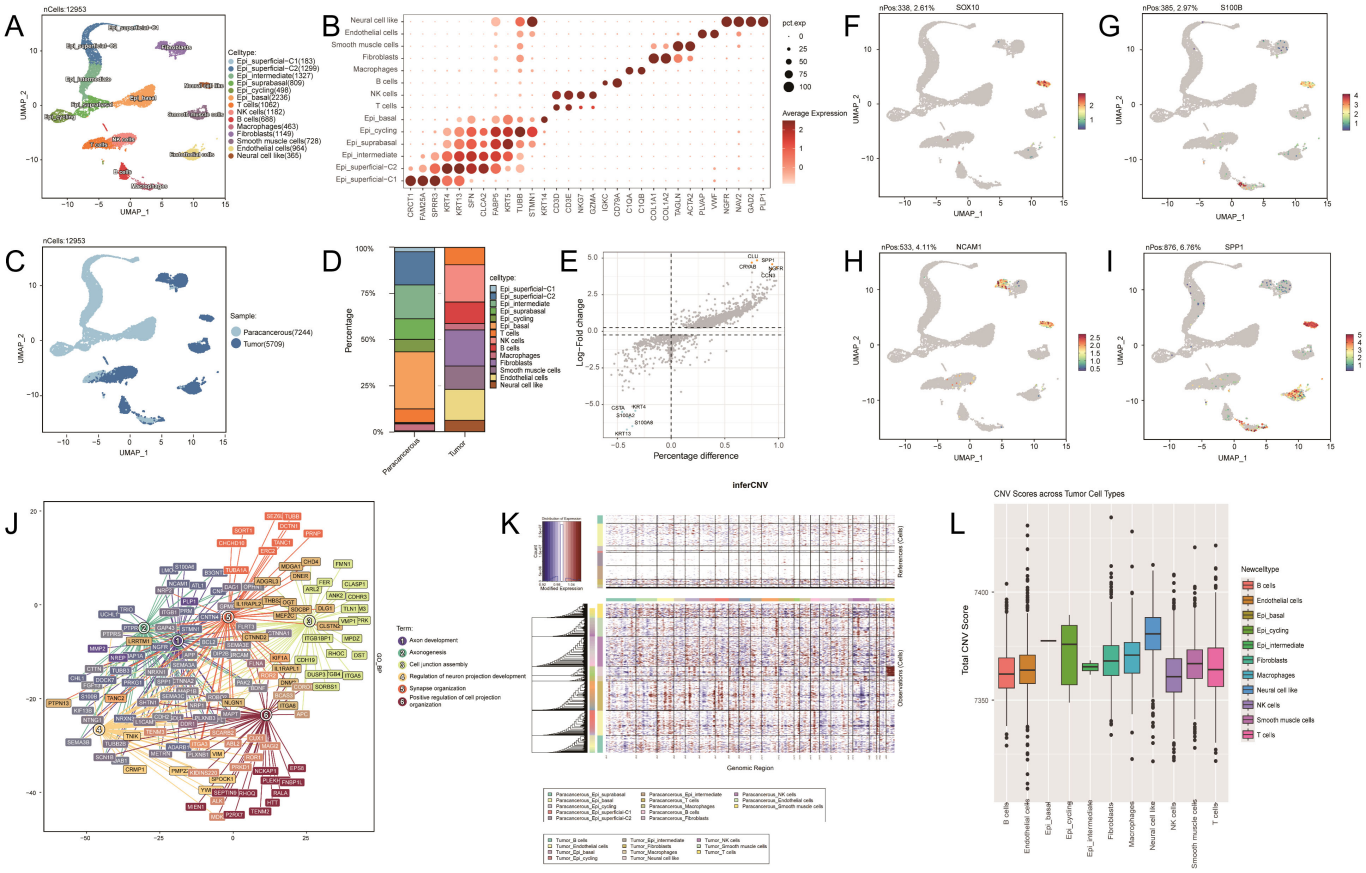
**(A)** Uniform Manifold Approximation and Projection (UMAP) representation of 14 identified cell types. N=12,953 cells. **(B)** Dot plot of the gene expression level of marker genes across cells. **(C)** UMAP representation showing two sample types, paracancerous and tumor. **(D)** Bar plot showing the cell type composition in paraneoplastic and tumor samples. **(E)** Volcano plot showing differential gene expression analysis for the neural cell like cluster. **(F–I)**: UMAP plots showing the expression levels of SOX10, S100B, NCAM1, and SPP1 across different cell clusters. **(J)** Gene Ontology analysis of biological processes for the neural cell-like cluster. **(K)** Heatmap showing large-scale copy number variations (CNVs) profile of each tumor cell cluster. **(L)** Box plot showing the CNV scores across different tumor cell types.

In this study, we used single-cell RNA-sequencing (scRNA-seq) to analyze cell clusters in tumor and paraneoplastic tissues. Following data quality control, 12,953 high-quality cells were obtained and analyzed using the UMAP clustering algorithm. We annotated 14 cell clusters, including neural cell-like cells (marker genes: NGFR, NAV2, GAD2, and PLP1), endothelial cells (marker genes: PLVAP and VWF), smooth muscle cells (marker genes: ACTA2 and PLVAP), and fibroblasts (marker genes: TAGLN and COL1A2) (**Figures 2A–B**). Significant differences were observed in cell composition between the tumor and paraneoplastic tissues (**Figure 2C**). Paraneoplastic tissues were mainly epithelial, while tumor tissues were more diverse, containing endothelial cells (16.7%), smooth muscle cells (12.6%), fibroblasts (19.5%), macrophages (3.4%), B cells (11.6%), NK cells (20.1%), and T cells (9.3%). Neural cell-like clusters (6.4%) were enriched in tumor tissues (**Figure 2D**) and further analyzed the subpopulations of these neural cell-like clusters. Differentially expressed genes (DEGs) analysis identified key genes (SOX10, SOX4, S100B, NCAM1, SPP1, NGFR, IGFBP5, STMN1, RUNX2, and TGFBI) (**Figure 2E**), with expression levels compared across clusters (**Figures 2F–I**). Gene Ontology and Kyoto Encyclopedia of Genes and Genomes analyses highlighted pathways like cell leading edge, cell–substrate junction, oxidative phosphorylation, and axon development in neural cell-like clusters (**Figure 2J**). Copy number variation (CNV) analysis revealed higher CNV levels in neural cell-like clusters, particularly in the X chromosome region (**Figures 2K–L**).

## 4 Discussion

The clinical presentation of esophageal granular cell tumors is nonspecific, and they are mostly observed incidentally during endoscopic examinations. Esophageal granulosa cell tumors (EGCT) often appear as submucosal protrusive lesions or sessile polyps on endoscopy and can be easily misdiagnosed as polyps or esophageal smooth muscle tumors. Significant esophageal granular cell tumors may result in esophageal stricture, retrosternal discomfort, or dysphagia.

The treatment of esophageal cell tumors should encompass a thorough evaluation of factors such as size, location, depth of invasion, and the individual patient’s clinical circumstances. Some researchers state that patients who are asymptomatic or are mildly symptomatic with tumors measuring <1 cm can be managed through regular monitoring. Conversely, other scholars advocate primary tumor resection as the preferred treatment for GCT, emphasizing the importance of endoscopic ultrasonography for pretreatment assessment. For tumors limited to the mucosal layer or with minimal submucosal infiltration and a size of <2 cm, Endoscopic Mucosal Resection or Endoscopic Submucosal Dissection is recommended. In cases of extensive submucosal infiltration, invasion of the lamina propria, or tumors >2 cm, complete surgical resection is recommended. Long-term postoperative follow-up is essential to monitor tumor recurrence and potential complications.

Free-flap repair of esophageal defects following surgery for giant GCT in the cervical esophagus is uncommon. In 2006, a single case involving the use of a forearm radial free flap for the reconstruction of a 5-cm defect in the cervical esophagus was reported (11). The patient underwent a one-year follow-up period, during which they exhibited normal diet intake, absence of dysphagia, normal speech, and a regular lifestyle. There is no consensus on the method of esophageal reconstruction following partial esophagectomy for benign esophageal lesions such as GCT. Currently, the methods used to reconstruct the esophagus include gastric pull-up (GPU), colonic interposition (CI), jejunal flap (JF), and myocutaneous or fasciocutaneous free flaps (MC/FCFFs). GPUs are widely used for esophageal reconstruction because of their rich blood supply and the advantage of requiring only a single anastomosis. However, reflux esophagitis, mechanical obstruction, dumping syndrome, and aspiration pneumonia are common postoperative complications associated with GPU (12, 13). Some surgeons prefer to use CI for esophageal reconstruction, which has fewer complications associated with gastric reflux than gastric pull-ups and is mostly used for esophageal reconstruction after resection of tumors in the upper third of the esophagus (13). Overall complication rates for CI range from 40 to 60%, with most mortality rates <10% (14). Conversely, studies have indicated that JF outperforms CI in various aspects, including the graft necrosis rate, anastomotic leakage incidence, length of hospital stay, and postoperative weight loss (15, 16). However, a limitation of JF is its limited ability to address extensive esophageal defects owing to its limited vascular mesenteric arcades (15, 16). The aforementioned esophageal reconstruction methods necessitate transabdominal surgery, which is unsuitable for esophageal reconstruction after local resection of benign cervical esophageal tumors, posing the risks of intestinal obstruction and delayed transoral feeding in the postoperative phase.

The advantages of MC/FCFFs for esophageal reconstruction include no requirement for transabdominal surgery, a low incidence of ischemic graft necrosis, and no catheter redundancy. However, the drawbacks are a high rate of anastomotic leakage and short food transport time (13). One study suggested that multilayer closure could reduce the incidence of fistulae (17). In previous clinical practice, MC/FCFFs were selected only when esophageal reconstruction via gastrointestinal catheterization failed. MC/FCFFs are often used as an option for the secondary repair of esophageal defects because secondary repair is often accompanied by the risks of enlarged tissue damage and poor recipient vascularization. Sturdy pedicle flaps are a safer alternative with a lower incidence of flap necrosis, fistulae, and strictures (18). The preferred application of MC/FCFF to repair esophageal defects after local esophageal resection has occasionally been reported. In 2015, Mohammad et al. reported a case of primary early squamous cell carcinoma of the cervical esophagus reconstructed by local resection with a radial forearm free flap (19). The patient maintained good esophageal function for almost 4 years after resection without any signs of recurrence. In 2024, a study reported six cases of cervical esophageal defect reconstruction using pedicle flaps without flap necrosis, esophageal fistula, hematoma, or wound dehiscence (20). Although MC/FCFFs for the repair of localized esophageal defects have demonstrated very reliable efficacy, their clinical application in esophageal reconstructive surgery has been limited because of obscuring of the tracheal structure, the high variability of vascular anatomy, and the highly demanding surgeon’s microvascular anastomosis technique.

Free flap esophageal reconstruction, a more complex surgery requiring intensive postoperative monitoring, offers superior reliability in terms of coverage capacity, tissue resistance, and distance management from the recipient vessel. We recommend that thin flaps be considered when reconstructing cervical esophageal defects after local resection of benign tumors. Surgeons should consider their expertise and adopt a customized, multidisciplinary approach to manage such cases.

To date, to our knowledge, we have found no published data on single-cell analysis of EGCT. To further understand the specific cellular status of esophageal granular cell tumors, we analyzed the differences in cell clusters between the tumor and paraneoplastic tissues of patients via scRNA-seq. The results revealed significant cellular heterogeneity in EGCT, characterized by a diversity of cell types, particularly enriched with the neural cell-like clusters (**Figure 2A**). Our findings are consistent with those of previous studies on the characteristics of its pathological origin, further supporting its neurogenic pathological features (1). By comparing the expression levels of DEGs, we observed that *SOX10*, *S100B*, and *NCAM1* (CD68) were significantly upregulated in the neural cell-like cluster (**Supplementary Figure 1F–H**), which is consistent with the findings of previous studies (1, 21). Our research further delineated the specific cell types that exhibited positive immune responses to these markers in GCT tissues and validated their neurogenic pathological features at single-cell resolution. Furthermore, *SPP1*, *NGFR*, *IGFBP5*, *STMN1*, *RUNX2*, and *TGFBI* were highly expressed in the neural cell-like cluster (**Figure 2I**; **Supplementary Figures 1A–F**). These genes are involved in the activation of signaling pathways related to tumor growth, metastasis, and immune evasion. Notably, *SPP1* is overexpressed in various cancers, promoting malignant progression and correlating with poor prognosis by enhancing cell survival, proliferation, and angiogenesis (22, 23). In multiple cancers, including non-small cell lung cancer, *STMN1* is abnormally expressed at high levels and promotes tumor cell proliferation by influencing microtubule stability and regulating phosphorylation levels (24–26). *RUNX2* plays a critical role in tumor progression and bone metastasis (27, 28), whereas *TGFBI* may contribute to immune evasion in the tumor immune microenvironment (29). Therefore, the high expression of genes identified in the neural cell-like cluster of the patient’s tumor tissue may indicate that this cluster plays an important role in the progression and malignancy of *EGCT*. Furthermore, CNV analysis revealed a significant increase in CNVs in the X-chromosome region of the neural cell-like cluster. This finding is consistent with that of previous research indicating a higher incidence of EGCT in females (30). The increase in CNVs on the X chromosome may represent a potential mechanism for the higher incidence of this tumor in females, and the results of this study suggest that sex differences play a significant role in the occurrence and progression of this tumor. This article emphasizes two aspects: the selection of flaps for esophageal defect repair and scRNA-seq technology. Both are aimed at providing clinical guidance for EGCT patients, with the ultimate goal of enhancing their diagnostic and therapeutic outcomes.

In summary, we present a rare case of a giant EGCT in the cervical segment that was analyzed for the first time using scRNA-seq. Assessment of the malignant potential of a tumor is the key to its management. Clinicians should consider the clinical presentation, choice of treatment options, and prognostic follow-up of esophageal GCT. We strongly recommend the early radical surgical resection of esophageal tumors with potentially malignant features. The MCFFs is an effective and reliable technique for reconstructing esophageal function post-surgery for esophageal tumors. Our patient had significantly reduced dysphagia, good nutritional status, normal phonation, and no postoperative complications such as fistulas or gastrointestinal stenosis. However, long-term follow-up and further statistical comparisons with other modalities for the reconstruction of esophageal function are needed to repair esophageal defects with MCFFs.

## 5 Conclusion

We strongly recommend the early radical surgical resection of esophageal tumors with potentially malignant features, and the MCFF is an effective and reliable technique for reconstructing esophageal function post-surgery for esophageal tumors.

## Data Availability

The raw data supporting the conclusions of this article will be made available by the authors, without undue reservation.

## References

[1] Abrikossoff A Myome Uber. Virchows Archiv fur Pathologische Anatomie und Physiologie und fur Klinische Medizin. (1926) 260:215–33. doi: 10.1007/BF02078314

[2] Lack EE Worsham RGF Callihan MD Crawford BE Klappenbach S Rowden G. Granular cell tumor: A clinicopathologic study of 110 patients. J Surg Oncol. (1980) 13:301–16. doi: 10.1002/jso.2930130405, PMID: 6246310

[3] Johnston MJ Helwig EB. Granular cell tumors of the gastrointestinal tract and perianal region A study of 74 cases. Digest Dis Sci. (1981) 26:807–16. doi: 10.1007/bf01309613, PMID: 6169495

[4] An S Jang J Min K Kim M Park H Park YS. Granular cell tumor of the gastrointestinal tract: histologic and immunohistochemical analysis of 98 cases. Hum Pathol. (2015) 46:813–9. doi: 10.1016/j.humpath.2015.02.005, PMID: 25882927

[5] Piecuch J Wiewiora M Latos W. Surgical treatment of a rare case of granular cell tumour of the cervical oesophagus. Videosurgery Miniinv. (2013) 8(2):166–9. doi: 10.5114/wiitm.2011.32819, PMID: 23837102 PMC3699767

[6] Loo CKC Santos LD Killingsworth MC. Malignant oesophageal granular cell tumour: a case report. Pathology. (2004) 36:506–8. doi: 10.1080/00313020412331282744, PMID: 15370125

[7] Audag N Toussaint M Prigent H Reychler G. Interpretation of Sydney Swallow Questionnaire results using the oropharyngeal dysphagia risk matrix. Neurogastroenterol Motility. (2024) 36(11):e14916. doi: 10.1111/nmo.14916, PMID: 39301584

[8] O’Neil KH Purdy M Falk J Gallo L. The dysphagia outcome and severity scale. Dysphagia. (1999) 14:139–45. doi: 10.1007/PL00009595, PMID: 10341109

[9] Ribeiro VV Batista DDJ Silveira WL Barbosa I Casmerides MCB Dornelas R. Reliability, measurement error, and responsiveness of the voice handicap index: A systematic review and meta-analysis. J Voice. (2024). 18:S0892-1997(24)00169-3. doi: 10.1016/j.jvoice.2024.05.017, PMID: 39030149

[10] Narasimhan SV Puttegowda K Sahana K. Adaptation and validation of the voice-related quality of life measure into kannada. J Voice. (2022) 39(2):568.e1–568.e6. doi: 10.1016/j.jvoice.2022.09.011, PMID: 36270921

[11] Marin VP Yu P Weber RS. Isolated cervical esophageal reconstruction for rare esophageal tumors. Head Neck. (2006) 28:856–60. doi: 10.1002/hed.20442, PMID: 16835909

[12] Cheng B Chang S Mao Z Li M Huang J Wang Z. Surgical treatment of giant esophageal leiomyoma. World J gastroenterology: WJG. (2005) 11:4258–60. doi: 10.3748/wjg.v11.i27.4258, PMID: 16015702 PMC4615455

[13] Merritt RE. Conduit selection for reconstruction after esophagectomy for esophageal cancer. Surg Oncol Clin N Am. (2024) 33:549–56. doi: 10.1016/j.soc.2024.01.001, PMID: 38789197

[14] Sanchez MV Alicuben ET Luketich JD Sarkaria IS. Colon interposition for esophageal cancer. Thorac Surg Clin. (2022) 32:511–27. doi: 10.1016/j.thorsurg.2022.07.006, PMID: 36266037

[15] Doki Y Okada K Miyata H Yamasaki M Fujiwara Y Takiguchi S. Long-term and short-term evaluation of esophageal reconstruction using the colon or the jejunum in esophageal cancer patients after gastrectomy. Dis Esophagus. (2008) 21:132–8. doi: 10.1111/j.1442-2050.2007.00738.x, PMID: 18269648

[16] Hung P Chen H Tu Y Kao Y. A comparison of different types of esophageal reconstructions: A systematic review and network meta-analysis. J Clin Med. (2022) 11:5025. doi: 10.3390/jcm11175025, PMID: 36078955 PMC9457433

[17] Tan NC Lin PY Kuo PJ Tsai YT Chen YC Nguyen KT. An objective comparison regarding rate of fistula and stricture among anterolateral thigh, radial forearm, and jejunal free tissue transfers in circumferential pharyngo-esophageal reconstruction. Microsurg. (2015) 35:345–9. doi: 10.1002/micr.22359, PMID: 25430852

[18] Ramella V Ferrari A Novati FC Arnež ZM Marchi G Rodda A. Secondary microsurgical reconstruction of the cervical esophagus: safer flaps and practical tips in a challenging situation. J Clin Med. (2024) 13:2726. doi: 10.3390/jcm13092726, PMID: 38731255 PMC11084327

[19] Ali Mohammad FHM Go PM Ghanem TM Stachler RM Hammoud ZM. Long-term survival after local resection of cervical esophageal cancer. Ann Thorac surgery. (2015) 99:2202–3. doi: 10.1016/j.athoracsur.2014.08.050, PMID: 26046877

[20] Agarwal A Philips R Fiorella M Amin DR Krein H Heffelfinger R. Complications and functional outcomes after esophageal reconstruction with an intact larynx. Laryngoscope. (2024) 134:1227–33. doi: 10.1002/lary.31055, PMID: 37712564

[21] Malik F Bernieh A Saad AG. Esophageal granular cell tumor in children: A clinicopathologic study of 11 cases and review of the literature. Am J Clin Pathol. (2023) 160:106–12. doi: 10.1093/ajcp/aqad025, PMID: 37026754

[22] Wei J Chen Z Hu M He Z Jiang D Long J. Characterizing intercellular communication of pan-cancer reveals SPP1+ Tumor-associated macrophage expanded in hypoxia and promoting cancer Malignancy through single-cell RNA-seq data. Front Cell Dev Biol. (2021) 2021:9. doi: 10.3389/fcell.2021.749210, PMID: 34676217 PMC8523849

[23] Lu D Yeh W Huang S Tang C Lin H Chou S. Osteopontin increases heme oxygenase–1 expression and subsequently induces cell migration and invasion in glioma cells. Neuro-Oncology. (2012) 14:1367–78. doi: 10.1093/neuonc/nos262, PMID: 23074199 PMC3480271

[24] Rubin CI Atweh GF. The role of stathmin in the regulation of the cell cycle. J Cell Biochem. (2004) 93:242–50. doi: 10.1002/jcb.20187, PMID: 15368352

[25] Iancu-Rubin C Atweh GF. p27Kip1 and stathmin share the stage for the first time. Trends Cell Biol. (2005) 15:346–8. doi: 10.1016/j.tcb.2005.05.008, PMID: 15951178

[26] Zeng L Lyu X Yuan J Chen Y Wen H Zhang L. STMN1 promotes tumor metastasis in non-small cell lung cancer through microtubule-dependent and nonmicrotubule-dependent pathways. Int J Biol Sci. (2024) 20:1509–27. doi: 10.7150/ijbs.84738, PMID: 38385074 PMC10878155

[27] Yin X Teng X Ma T Yang T Zhang J Huo M. RUNX2 recruits the NuRD(MTA1)/CRL4B complex to promote breast cancer progression and bone metastasis. Cell Death Differentiation. (2022) 29:2203–17. doi: 10.1038/s41418-022-01010-2, PMID: 35534547 PMC9613664

[28] Lin T. RUNX2 and cancer. Int J Mol Sci. (2023) 24:7001. doi: 10.3390/ijms24087001, PMID: 37108164 PMC10139076

[29] Lecker LSM Berlato C Maniati E Delaine-Smith R Pearce OMT Heath O. TGFBI production by macrophages contributes to an immunosuppressive microenvironment in ovarian cancer. Cancer Res. (2021) 81:5706–19. doi: 10.1158/0008-5472.CAN-21-0536, PMID: 34561272 PMC9397609

[30] Polasek JB Laviv Y Nigim F Rojas R Anderson M Varma H. Granular cell tumor of the infundibulum: a systematic review of MR-radiography, pathology, and clinical findings. J Neuro-Oncol. (2018) 140:181–98. doi: 10.1007/s11060-018-2986-2, PMID: 30141059

