# Supplementary material for: Case Report: A large granular cell tumor of the cervical esophagus with single cell RNA sequencing analysis
Source: Front Oncol. 2025 Sep 2;15:1580121. doi: 10.3389/fonc.2025.1580121 (PMC12436342; doi:10.3389/fonc.2025.1580121)
Supplement: Supplementary file 1 [file DataSheet1.pdf]

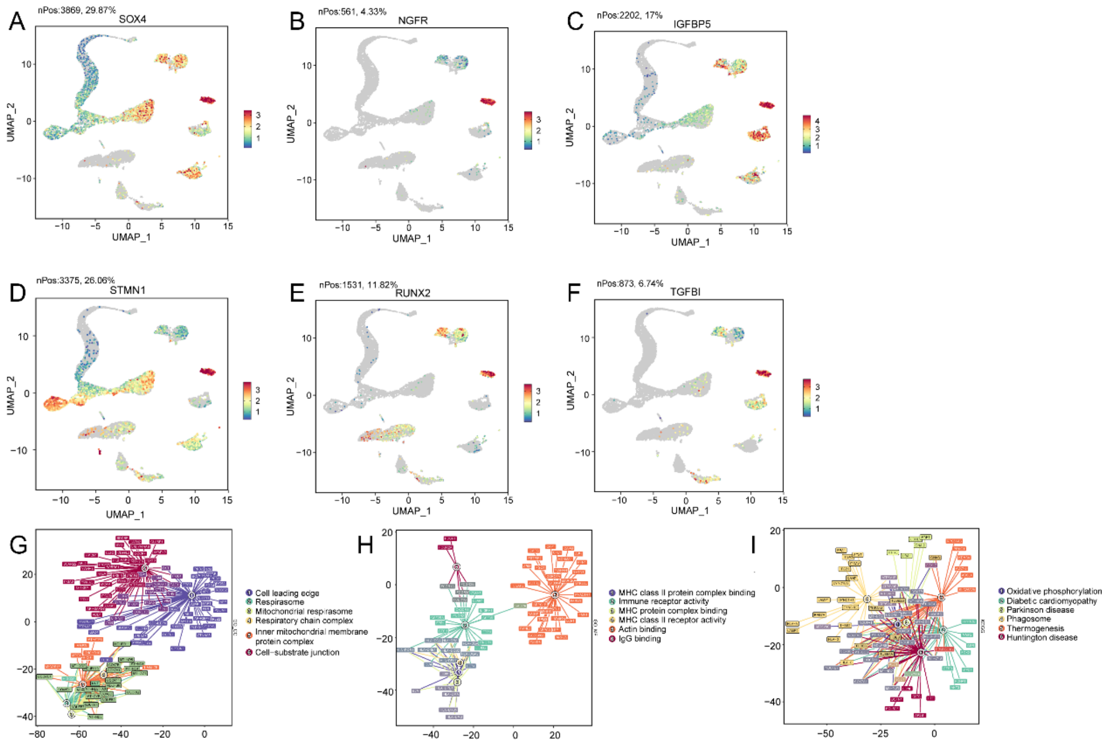

Supplementary Fig. 1 A-F): Uniform Manifold Approximation and Projection (UMAP) plots showing the expression levels of SOX4, NGFR, IGFBP5, STMN1, RUNX2, and TGFBI across different cell clusters. G-H) Gene Ontology (GO) analysis for the neural cell-like cluster. I): Kyoto Encyclopedia of Genes and Genomes (KEGG) pathway analysis for the neural cell-like cluster.
